# ﻿Mitogenomes of the two historical species *Seiraferrarii* Parona, 1888 and *Seirapallidipes* Reuter, 1895 (Collembola, Entomobryidae, Seirinae) with their phylogenetic placement within Seirinae

**DOI:** 10.3897/zookeys.1176.108859

**Published:** 2023-08-24

**Authors:** Nerivania Nunes Godeiro, Yun Bu, Daniel Winkler

**Affiliations:** 1 Natural History Research Center, Shanghai Natural History Museum, Shanghai Science & Technology Museum, Shanghai, 200041, China Shanghai Natural History Museum, Shanghai Science & Technology Museum Shanghai China; 2 University of Sopron, Faculty of Forestry, Institute of Wildlife Biology and Management, Bajcsy-Zs. str. 4, H–9400, Sopron, Hungary University of Sopron Budapest Hungary

**Keywords:** Entomobryoidea, Europe, gene order, Hungary, mitochondrial genomes, phylogeny, springtails

## Abstract

The present paper reports the first occurrence of *Seiraferrarii* Parona, 1888 from Hungary. On this occasion, molecular analyses were performed on both *S.ferrarii* and another historical species of the genus, *S.pallidipes* Reuter, 1895, originally described from Hungary. Using low-coverage whole-genome sequencing, the complete mitogenomes were assembled and annotated using MitoZ. To test the phylogenetic placement of both species, we performed maximum likelihood and Bayesian analyses using a matrix containing 14 Seirinae species and two outgroups. Both resultant trees showed that the European populations of the sampled *Seira* spp. likely derive from ancestral branches of Seirinae, compared to the Asian and American populations. Our results put in question the monophyly of the genus *Seira*, as already observed in previous studies.

## ﻿Introduction

Although *Seira* Lubbock, 1870 is one of the most widespread genera of Entomobryidae worldwide, its high species richness is mainly found in the tropics (e.g., Mari Mutt 1986; Cipola et al. 2014, 2018b; Godeiro and Bellini 2014), with relatively few representatives occurring in Europe, most of which found in Mediterranean countries, under subtropical climates (e.g., Gama 1964; Tosi and Parisi 1990; Cipola et al. 2018a).

*Seiraferrarii* Parona, 1888 was originally described from Italy (Genova), and later also recorded in the Spanish mainland (Yosii 1959), Bulgaria, French mainland, Greek mainland, Malta, Republic of Moldova, Spain, Portugal, Romania, various islands (Canary Is., Corsica, Dodecanese Is., Sicily) (Bedos 2023), North Africa (Jacquemart and Jacques 1980; Barra 2004), and Israel (Gruia et al. 2000). From the capital of Hungary, Loksa and Loksa (1994) reported the presence of a “particular” *Seira* sp. juv., which however remained undetermined. On revisiting the sampling site, we managed to collect several specimens of this abovementioned *Seira* sp., which were identified as *S.ferrarii*, and thus represent the first occurrence of the species in Hungary.

*Seirapallidipes* Reuter, 1895, on the other hand, was originally described from Hungary, and was recently redescribed and synonymized with *S.pillichi* Stach, 1929 by Winkler and Dányi (2017). Outside Hungary, the species occurs only in Austria and Serbia (Bedos 2023).

*Seiradollfusi* Carl, 1899 is a widely distributed species in Europe (Bedos 2023). However, as its true identity is unclear (the species has not yet been redescribed in detail based on type material or specimens collected at the type locality), we refer to specimens collected in Hungary for our study as S.cf.dollfusi.

The main goal of our study was to investigate for the first time the phylogenetic position of three European species of Seirinae. Previous studies focused on the Neotropical region (Godeiro et al. 2020) and the most embracing was recently published, containing species from the Neotropics and Asia (Godeiro et al. 2023). The internal organization of the subfamily is uncertain, and extensive taxon sampling is currently underway to propose a global phylogenetic study with dispersion routes and possible new genera.

## ﻿Materials and methods

Specimens of *S.ferrarii* were collected in a xerophilous dolomite-steppe meadow association, Tétényi Plateau, Budapest, Hungary (47°25'6"N, 18°56'58"E, 195 m a.s.l.) on 7.X.2022 (leg. D. Winkler & M. Korda), while specimens of *S.pallidipes* were collected in a secondary hay meadow, Sopron, Hungary (47°45'31"N, 16°36'58"E, 169 m a.s.l.) on 4.VI.2022 (leg. D. Winkler). Seiracf.dollfusi was sampled on a calcareous open rocky grassland, Zuppa-tető, Szárliget, Hungary (47°30'10"N, 18°30'52.41"E, 359 m) on 6.IX.2022 (leg. D. Winkler).

On each occasion, an entomological aspirator was used for collection. Specimens were stored in absolute ethanol until further analysis. A Zeiss Stemi 508 stereomicroscope was used to sort the material. Habitus of the two studied species was photographed with a Canon EOS 7D digital camera attached to the stereomicroscope using a C-mount adapter.

Specimens for morphological identification were cleared using Nesbitt’s fluid and then mounted on permanent slides in Hoyer’s medium, following the protocol described by Jordana et al. (1997). The slides were examined under a Leica DM2500 LED microscope with conventional bright light and phase contrast.

Part of the material preserved in absolute ethanol was sent to Shanghai Yaoen Biotechnology Co., Ltd, China, where all laboratory experiments, including DNA extraction, amplification, and library construction were made according to the procedures suggested by the kits manufacturers. For DNA extraction, the TIANamp MicroDNA extraction kit (Tiangen Co., Ltd, China) was used. Libraries were constructed using KAPA Hyper Prep Kit (Roche, Basel, Switzerland). Approximately 10 Gbp of paired-end reads from each species were sequenced by an Illumina NovaSeq 6000 platform. This amount of data was enough to have a good coverage to assemble complete mitogenomes.

Mitogenomes were assembled, annotated, and visualized using MitoZ v.2.4-alpha (Meng et al. 2019). A manual check was necessary to confirm the start and end points of protein-coding genes (PCGs). Mitochondrial genome sequences with annotations and raw sequence data were submitted to the NCBI nucleotides and SRA databases (https://www.ncbi.nlm.nih.gov/), with project number PRJNA971781. To complete our phylogenetic dataset, protein-coding genes (PCG’s) of another 11 species of Seirinae and two Lepidocyrtinae (outgroups) were downloaded from NCBI (Table 1). The partial mitogenome of S.cf.dollfusi (9298 bp), also sequenced and assembled during the present study, was included in the phylogenetic analyses to test its closer relationship with *S.pallidipes*. The sequence is publicly available at: https://doi.org/10.6084/m9.figshare.23654097.

**Table 1.** Taxonomical information of the species used in the phylogenetic analyses. The newly assembled mitogenomes are represented in bold. *Mitogenome partially recovered. NA: not applicable.

	Species	Subfamily	Country	GenBank number
1	*Lepidocyrtusfimetarius* Gisin, 1964	Lepidocyrtinae	China	NC_047189.1
2	*Lepidocyrtussotoi* Bellini & Godeiro, 2015	Lepidocyrtinae	Brazil	MT928545.1
3	*Lepidocyrtinusdapeste* Santos & Bellini, 2018	Seirinae	Brazil	MF716609.1
4	*Lepidocyrtinusharena* (Godeiro & Bellini, 2014)	Seirinae	Brazil	MF716617.1
5	*Seiraatrolutea* (Arlé, 1939)	Seirinae	Brazil	MF716602.1
6	*Seiraboneti* (Denis, 1948)	Seirinae	China	OP181099.1
7	*Seirabrasiliana* (Arlé, 1939)	Seirinae	Brazil	MF716619.1
8	*Seiradowlingi* (Wray, 1953)	Seirinae	Brazil	MF716615.1
9	Seiracf.dollfusi*	Seirinae	Hungary	NA
10	***Seiraferrarii***	Seirinae	Hungary	OR206048.1
11	***Seirapallidipes***	Seirinae	Hungary	OR115504.1
12	*Seiraritae* Bellini & Zeppelini, 2011	Seirinae	Brazil	MF716616.1
13	*Seirasanloemensis* Godeiro & Cipola, 2020	Seirinae	Cambodia	MT997754.1
14	*Seiratinguira* Cipola & Bellini, 2014	Seirinae	Brazil	MF716620.1
15	*Tyrannoseirabicolorcornuta* (Bellini, Pais & Zeppelini, 2009)	Seirinae	Brazil	MF716599.1
16	*Tyrannoseiraraptora* (Zeppelini & Bellini, 2006)	Seirinae	Brazil	MF716610.1

Previously to the alignment, nucleotide sequences of the 13 PCGs of the 16 species that comprise our phylogenetic dataset were translated into amino acids using TransDecoder v.5.5.0 (https://github.com/TransDecoder/TransDecoder). Previous phylogenetic studies of Collembola showed that due to the high heterogeneity of mitochondrial sequences, matrices created using amino acids produce better results than using nucleotides (Bellini et al. 2023; Godeiro et al. 2021). Also, according to a recent analysis, site-wise heterogeneity is typically a more significant source of bias in phylogenomic inference than protein-wise heterotachy (Wang et al. 2019; Yu et al. 2022). Independent files containing the PCGs were aligned by MAGUS (Smirnov and Warnow 2021) employing MAFFT (Katoh et al. 2019). An automated alignment trimming was performed by BMGE v.1.12 (Criscuolo and Gribaldo 2010). PhyKIT v.1.9.0 (Steenwyk et al. 2021) concatenated the alignments and generated the partition scheme. The final matrix had 3104 amino acid sites and 13 loci.

To test the phylogenetic placement of the European species of *Seira*, we performed two phylogenetic inferences. IQ-Tree v.2.0.7 (Minh et al. 2020) was used to make the maximum likelihood (ML) analyses with 1000 ultrafast bootstrap replicates (Hoang et al. 2018) and SH-aLRT support (Guindon et al. 2010). The best model for each partition was suggested by ModelFinder (Kalyaanamoorthy et al. 2017). The partitions and models used are listed in Table 2. The Bayesian inference (BI) was performed using PhyloBayes-MPI v.1.8 (Lartillot et al. 2013), default model CAT+GTR with four rate categories, discretized gamma distribution of rates across sites, sampling every 100 generations, with the first 1000 sampled trees discarded as “burn-in”. Two Markov Chain Monte Carlo (MCMC) chains were run until the likelihood had satisfactorily converged (maxdiff < 0.3). Phylogenetic trees were visualized in Figtree v.1.3.1 (Rambaut 2016).

**Table 2.** Partitioning scheme and substitution models selected by ModelFinder used for maximum likelihood analyses.

Partition	Genes	Model
1	ATP6 / NAD1 / ND4L	mtART
2	ATP8 / ND2 / ND6	mtART+F
3	COX1/ COX2 / COX3 / CYTB	mtART
4	ND3 / ND4 / ND5	mtART

## ﻿Results

**Figure 1.**
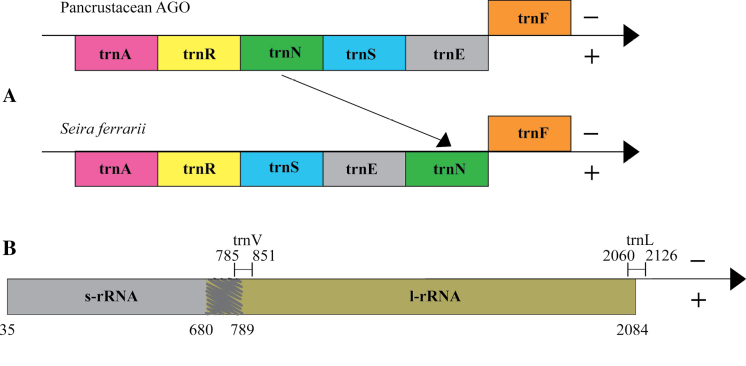
**A** gene order comparison between Pancrustacean and *Seiraferrarii* (clockwise direction) **B** region comprising rRNAs of *S.ferrarii* mitogenome (anti-clockwise direction). Scratch indicates the overlap between genes. Numbers represent the location of the genes in base pairs.

**Figure 2.**
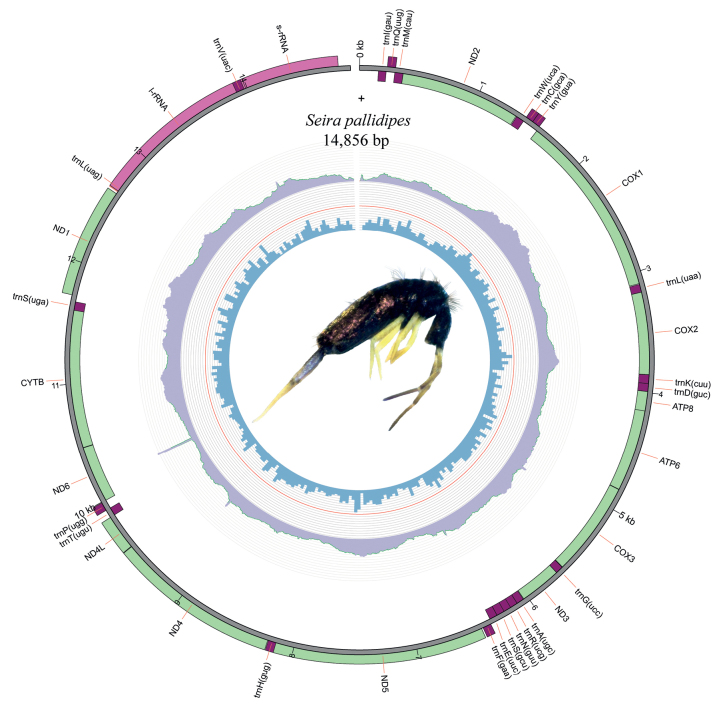
Circular representation of the mitogenome of *Seirapallidipes*. The innermost circle shows the GC content; the middle circle shows the reads coverage, and the outermost circle shows the gene features, rRNA, tRNA, and CDS. Photo in the center represents the original coloration of a specimen preserved in ethanol.

**Figure 3.**
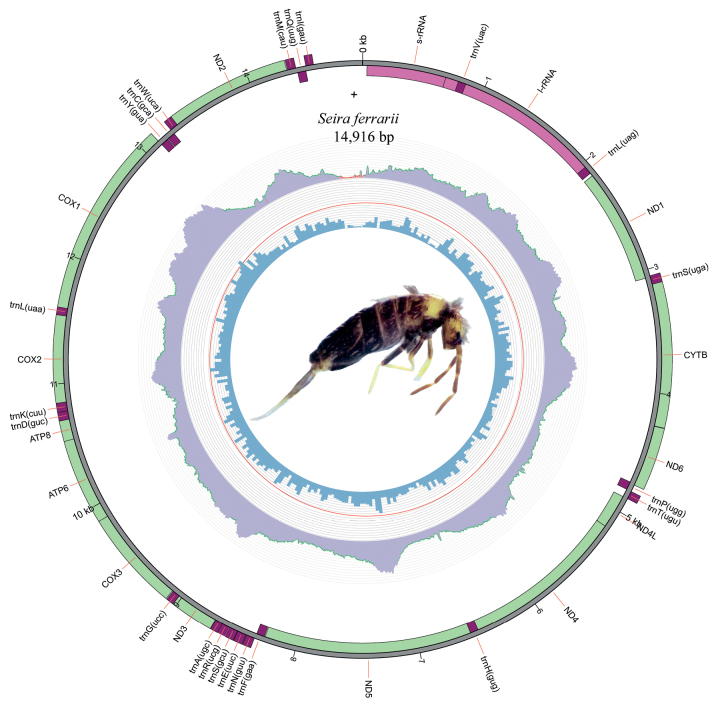
Circular representation of the mitogenome of *Seiraferrarii*. The innermost circle shows the GC content; the middle circle shows the reads coverage, and the outermost circle shows the gene features, rRNA, tRNA, and CDS. Photo in the center represents the original coloration of a specimen preserved in ethanol.

Mitochondrial genomes of *Seirapallidipes* and *S.ferrarii* have 14,856 bp and 14,916 bp in length, respectively (Figs 2, 3). All 13 PCGs, 2 rRNAs, and 22tRNAs were found. In *S.ferrarii*, the direction of transcription was anti-clockwise, and a rare gene translocation was observed in the region ranging from 8337 bp to 8550 bp. The order of tTNAs is normally tRNA-Asn (trnN) → tRNA-Ser 1 (trnS1) → tRNA-Glu (trnE), but in *S.ferrarii*, the gene order is tRNA-Ser 1 (trnS1) → tRNA-Glu (trnE) → tRNA-Asn (trnN), meaning that a translocation happened in the position of trnN (Figs 1, 3). For this reason, *S.ferrarii* does not present the Pancrustacean ancestral gene order, like most mitogenomes of Seirinae sequenced until now, including *S.pallidipes*. Another interesting characteristic observed during the mitogenome analysis were long overlaps between the genes tRNA-Leu (trnL), 16S ribosomal RNA (l-rRNA), tRNA-Val (trnV), 12S ribosomal RNA (s-rRNA), with trnV totally inserted on the l-rRNA sequence (Figs 1–3). These overlaps were observed in all Seirinae mitogenomes sequenced to date but have never been reported before. A broader study needs to be carried out to compare the gene order of other Collembola taxa to verify whether these differences have significant evolutionary importance and phylogenetic signals.

The most common start-stop codons in *S.pallidipes* were ATT/ATG-TAA, and in *S.ferrarii* ATT/ATA-TAA (Tables 3, 4). This pattern is the most common in Collembola mitogenomes (Godeiro et al. 2022). AT-rich regions have lengths of 185bp and 421bp in *S.pallidipes* and *S.ferrarii*, respectively. The nucleotide composition of both mitogenomes presented higher AT content than other Seirinae mitogenomes, 78% in *S.pallidipes* and 80% in *S.ferrarii*.

**Table 3.** Gene order and features of the mitochondrial genome of *Seirapallidipes*.

*Seirapallidipes* – 14,856 bp					
Start	End	Length(bp)	Direction	Start/End code	Gene product [gene name]
155	219	65	+		tRNA-Ile [trnI(gau)]
225	293	69	-		tRNA-Gln [trnQ(uug)]
290	359	70	+		tRNA-Met [trnM(cau)]
359	1354	996	+	ATG/TAA	NADH dehydrogenase subunit 2 [ND2]
1361	1429	69	+		tRNA-Trp [trnW(uca)]
1429	1492	64	-		tRNA-Cys [trnC(gca)]
1492	1558	67	-		tRNA-Tyr [trnY(gua)]
1563	3098	1536	+	ATT/TAA	cytochrom e c oxidase subunit I [COX1]
3105	3171	67	+		tRNA-Leu [trnL(uaa)]
3171	3851	681	+	ATA/TAA	cytochrome c oxidase subunit II [COX2]
3853	3925	73	+		tRNA-Lys [trnK(cuu)]
3925	3991	67	+		tRNA-Asp [trnD(guc)]
3991	4158	168	+	ATT/TAA	ATP synthase F0 subunit 8 [ATP8]
4152	4832	681	+	ATG/TAG	ATP synthase F0 subunit 6 [ATP6]
4835	5623	789	+	ATG/TAA	cytochrome c oxidase subunit III [COX3]
5627	5690	64	+		tRNA-Gly [trnG(ucc)]
5690	6034	345	+	ATT/TAA	NADH dehydrogenase subunit 3 [ND3]
6039	6100	62	+		tRNA-Ala [trnA(ugc)]
6100	6164	65	+		tRNA-Arg [trnR(ucg)]
6163	6228	66	+		tRNA-Asn [trnN(guu)]
6225	6292	68	+		tRNA-Ser 1 [trnS1(gcu)]
6292	6358	67	+		tRNA-Glu [trnE(uuc)]
6370	6439	70	-		tRNA-Phe [trnF(gaa)]
6452	8146	1695	-	ATA/TAA	NADH dehydrogenase subunit 5 [ND5]
8148	8213	66	-		tRNA-His [trnH(gug)]
8218	9567	1350	-	ATG/TAA	NADH dehydrogenase subunit 4 [ND4]
9571	9843	273	-	ATT/TAA	NADH dehydrogenase subunit 4L [ND4L]
9855	9925	71	+		tRNA-Thr [trnT(ugu)]
9925	9995	71	-		tRNA-Pro [trnP(ugg)]
9997	10467	471	+	ATT/TAA	NADH dehydrogenase subunit 6 [ND6]
10470	11609	1140	+	ATG/TAA	cytochrome b [CYTB]
11608	11674	67	+		tRNA-Ser 2[trnS2(uga)]
11675	12616	942	-	ATT/TAA	NADH dehydrogenase subunit 1 [ND1]
12617	12684	68	-		tRNA-Leu [trnL(uag)]
12651	14093	1443	-		16S ribosomal RNA [l-rRNA]
13908	13975	68	-		tRNA-Val [trnV(uac)]
13988	14758	771	-		12S ribosomal RNA [s-rRNA]
14778	107	185			AT-rich region

**Table 4.** Gene order and features of the mitochondrial genome of *Seiraferrarii*.

*Seiraferrarii* – 14,916 bp					
Start	End	Length (bp)	Direction	Start/End code	Gene name
35	789	755	+		s-rRNA
785	851	67	+		trnV(uac)
680	2084	1405	+		l-rRNA
2060	2126	67	+		trnL(uag)
2126	3064	939	+	TTG/TAA	ND1
3055	3126	72	-		trnS2(uga)
3125	4261	1137	-	ATG/TAA	CYTB
4264	4758	495	-	ATA/TAA	ND6
4749	4815	67	+		trnP(ugg)
4815	4879	65	-		trnT(ugu)
4881	5159	279	+	ATT/TAA	ND4L
5159	6502	1344	+	ATA/TAA	ND4
6502	6568	67	+		trnH(gug)
6568	8259	1692	+	ATT/T--	ND5
8267	8333	67	+		trnF(gaa)
8337	8406	70	-		trnN(guu)
8409	8481	73	-		trnE(uuc)
8483	8550	68	-		trnS1(gcu)
8553	8618	66	-		trnR(ucg)
8618	8678	61	-		trnA(ugc)
8680	9024	345	-	ATT/TAA	ND3
9025	9086	62	-		trnG(ucc)
9092	9880	789	-	ATG/TAA	COX3
9880	10557	678	-	ATG/TAA	ATP6
10551	10712	162	-	ATA/TAA	ATP8
10713	10780	68	-		trnD(guc)
10780	10851	72	-		trnK(cuu)
10851	11534	684	-	ATA/TAA	COX2
11535	11600	66	-		trnL(uaa)
11595	13133	1539	-	ATT/TAA	COX1
13135	13199	65	+		trnY(gua)
13205	13268	64	+		trnC(gca)
13267	13333	67	-		trnW(uca)
13333	14298	966	-	ATA/TGA	ND2
14316	14384	69	-		trnM(cau)
14390	14458	69	+		trnQ(uug)
14464	14527	64	-		trnI(gau)
14530	35	421			AT-rich region

**Figure 4.**
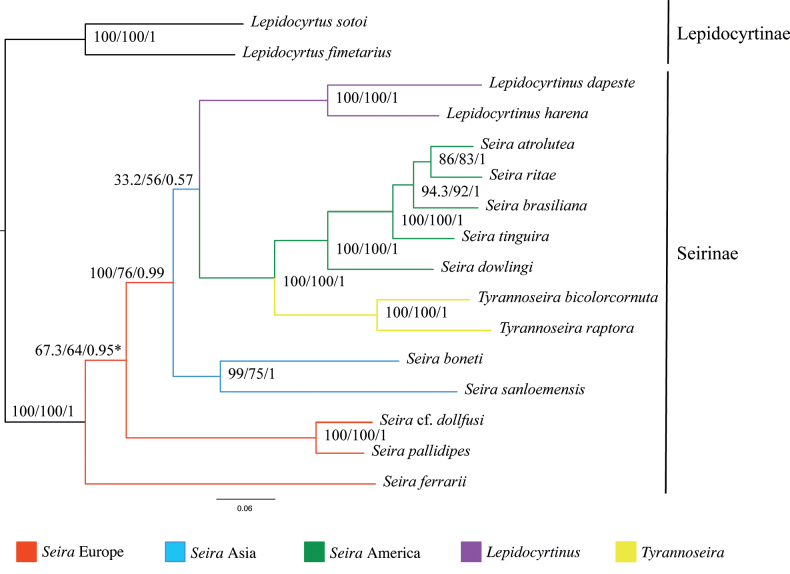
Phylogenetic placement of European *Seira*. Tree constructed based on maximum likelihood and Bayesian inferences (BI) from mitochondrial genomes. Numbers at the nodes represent the SH-aLRT support, bootstrap values (both for maximum likelihood), and the posterior probability (BI support), respectively. *In the BI the topology was: (S.cf.dollfusi + *S.pallidipes*) + *Seiraferrarii* + (other Seirinae).

Our phylogenetic results placed the European population of the sampled *Seira* spp. as ancestral to the Asian and American populations mostly with high SH-aLRT support and Bayesian posterior probability (Fig. 4). The geographic proximity on this scale can lead to genetic adaptation for specific conditions (e.g., different climate, habitat) and can result in a striking level of genetic differentiation within a genus, as observed in the case of *Seira*.

## ﻿Discussion

Most of the European *Seira* species are from the Mediterranean (e.g., Gama 1964; Gruia et al. 2000; Cipola et al. 2018a). As several phylogeographic studies and reviews have pointed out, the Mediterranean region (in particular, but not exclusively, Iberia, Italia, and the Balkans) was also important refugia for thermophilic species during glacial periods (e.g., Provan and Bennett 2008; Cicconardi et al. 2010; Costa et al. 2013; Fiera et al. 2017). High mountains ranging to the north, like the Alps or the Pyrenees, acted as barriers to dispersal and, thus, to gene flow, resulting in long-term isolated evolution and formation of new lineages (Gómez and Lunt 2007; Hewitt 2011; Fiera et al. 2017).

Of the studied species, *S.ferrarii* has the broadest distribution area, ranging from the Iberian Penisula to the Near East in the Mediterranean and also occurring in some Central-European countries. *Seirapallidipes*, on the other hand, has a very narrow known distribution, and the data so far suggest that its range is restricted to the Carpathian Basin. Collembola species with limited distribution have either a limited dispersal ability, very narrow habitat requirements, or their distributions are limited by geographical barriers (Fiera et al. 2017). As a part of an entirely epigeic group, *S.pallidipes* is also characterized by a strong dispersal ability, and its habitat requirements are not restrictive either (the species apparently occurs in various types of grasslands). Nevertheless, due to the geographically transitional position and the climatic conditions, the Carpathian Basin is rich in endemic and relict species (Varga 1995), and *S.pallidipes* may be one of its representatives.

*Seiraferrarii* represents a separate clade within Seirinae, while *S.pallidipes* with S.cf.dollfusi together form another clade (Fig. 4). The latter two species are more closely related morphologically by sharing a similar, mostly polichaetotic, dorsal macrochaetotaxy from Th II to Abd II, and are part of the “*squamoornata*-group” sensu Cipola et al. (2018a). On the other hand, *S.ferrarii* shows a clear reduction in dorsal macrochaetotaxy and thus was classified in the so-called “*domestica*-group” sensu Jacquemart (1974). This kind of division of species groups established based on the macrochaetotaxy distribution can be observed in other derived genera of Entomobryomorpha, most notably in *Lepidocyrtus* (Mateos et al. 2018; Winkler et al. 2020).

This study is part of a larger initiative to better understand the dispersion pathways of Seirinae species. Systematic research on this subfamily is impacted by the scarcity of specialists based in other continents, with the exception of South America. Despite the limited taxon sample, our phylogeny (Fig. 4) pointed out that the genus *Seira* is not monophyletic, possibly should be subdivided into more genera, and that the morphology of the subfamily needs to be more deeply investigated.

## ﻿Additional information

### Author ORCIDs

Nerivania Nunes Godeiro https://orcid.org/0000-0002-1669-6124

Yun Bu https://orcid.org/0000-0002-7177-9686

Daniel Winkler https://orcid.org/0000-0002-6008-0562

### Data availability

All accession numbers and links for the data that support the findings of this study are available in the main text.
